# Extra X, extra questions: Trisomy X syndrome and IgA deficiency – a case report

**DOI:** 10.3389/fimmu.2024.1518076

**Published:** 2024-12-06

**Authors:** Fabrizio Leone, Alessandra Gori, Bianca Laura Cinicola, Giulia Brindisi, Vittorio Maglione, Caterina Anania, Anna Maria Zicari

**Affiliations:** ^1^ Department of Maternal Infantile and Urological Sciences, Sapienza University of Rome, Rome, Italy; ^2^ Department of Translational and Precision Medicine, Sapienza University of Rome, Rome, Italy; ^3^ Department of Experimental Medicine, Sapienza University of Rome, Rome, Italy

**Keywords:** Trisomy x, IgA deficiency, immunodeficiency, Foxp3, CVID

## Abstract

While Trisomy X syndrome is typically characterized by developmental and cognitive variations, it is not commonly associated with immunodeficiencies. We report the unique case of a 6-year-old girl with Trisomy X presenting with selective IgA deficiency, challenging the conventional understanding of this chromosomal condition. The patient exhibited recurrent respiratory infections and gastrointestinal symptoms, evaluated in the context of her genetic background of Trisomy X and significantly low levels of IgA (0.03 g/L), yet normal IgG and IgM levels. Immunological assessment revealed a poor response to vaccination to HBV, necessitating an adapted vaccination strategy. Gastrointestinal investigations indicated paradoxical diarrhea secondary to chronic constipation, managed with dietary interventions. The presence of an extra X chromosome raises questions about the potential over-expression of genes that escape X-chromosome inactivation, such as *FOXP3*, which is crucial for the regulation of regulatory T cells. An abnormal expression of *FOXP3* could lead to either heightened immune regulation, increasing susceptibility to infections, or to immune dysregulation. Although Trisomy X is not typically associated with immunodeficiencies, this case, paralleled by another patient with Trisomy X and CVID, suggests a need for further speculative research into possible genetic links. Moreover, a 1969 study reported lower IgA levels in women with an extra X chromosome. In conclusion, this case aims to underscore the necessity for a deeper genetic and immunological evaluation in chromosomal anomalies like Trisomy X to fully understand their speculative impact on immune function.

## Introduction

Trisomy X is the most common female chromosomal abnormality; this condition marked by an additional X chromosome in females, traditionally focuses on developmental and cognitive outcomes (1). It is hypothesized that the phenotype observed in Trisomy X may be due to the overexpression of genes that are not subject to X-inactivation, although the specific relationships between genotype and phenotype are still unclear. While Triple X syndrome has typically not been associated with immunodeficiencies, a case (2) documents a patient with this syndrome who developed common variable immunodeficiency (CVID). This unique instance suggests that further research might be needed to explore potential immunological aspects of Triple X syndrome that have not been widely recognized in the medical literature. CVID, despite being a prevalent immunodeficiency characterized by hypogammaglobulinemia, does not have a well-defined genetic cause in many cases. The heterogeneity of its clinical presentation and the varying immunological defects observed complicate the identification of specific genetic factors. While certain monogenic causes have been identified in a subset of patients, the genetic underpinnings for most CVID cases remain elusive, suggesting a complex interplay of multiple genes and environmental factors rather than a single genetic origin (3).

CVID often begins as a more benign immunological disorder such as Selective IgA Deficiency (SIgAD), which is the most common primary immunodeficiency. SIgAD often presents with mild clinical manifestations, making it a potential precursor to more severe immunodeficiencies like CVID. Understanding this progression is crucial, especially considering that many individuals with SIgAD may initially exhibit only mild or no symptoms but are at a higher risk of evolving into conditions with broader immunological deficits (4). The relationship between hypogammaglobulinemia and the X chromosome is compellingly highlighted by evidence indicating that the serum levels of immunoglobulins, particularly IgM, vary according to the number of X chromosomes an individual possesses. This suggests a potential genetic basis on the X chromosome for the regulation of certain immunoglobulins. It is evident that X chromosome aneuploidies are significantly associated with immune deficiencies, particularly those involving immunoglobulins (5). This includes both structural and numerical anomalies, such as the additional X chromosome in conditions like Klinefelter Syndrome (47,XXY) and Trisomy X, or the single X chromosome in Turner Syndrome (45,X). The presence of specific X-linked genes that influence B cell function directly, such as those implicated in X-linked agammaglobulinemia (XLA) and Hyper-IgM Syndrome (HIGM), further illustrates the importance of the X chromosome in regulating immunoglobulin levels. These conditions show how mutations or numerical abnormalities in the X chromosome can lead to significant immunodeficiencies, marked by deficiencies in immunoglobulin production (5). This connection is crucial for understanding the broader implications of Trisomy X and similar aneuploidies on immune function, as these conditions can result in complex immune dysregulation, including immunoglobulin deficiencies. Trisomy 21 serves as a prominent example of aneuploidy linked to immune deficiency, primarily driven by overexpression of interferon receptors on chromosome 21, which leads to chronic immune activation (6). Additionally, studies highlight profound abnormalities in B-cell development, with reductions in transitional and mature B-cell subsets, further compromising immune function and increasing susceptibility to infections and autoimmune conditions (7). Notably, individuals with additional X chromosomes, such as those with the XXX karyotype, exhibit significantly higher levels of IgM, pointing towards a gene-dosage effect from the X chromosome that influences immune function (8). Despite the recognized role of the X chromosome in immunity, there is limited description in the literature regarding Trisomy X.

The presence of an extra X chromosome may disrupt the delicate balance of immune regulation, potentially through mechanisms that escape traditional genetic inactivation processes, leading to varied immune responses. We present the case of a patient with Triple X syndrome and SIgAD, speculating on the possible links between this genetic condition and immunodeficiencies.

## Case presentation

**Figure 1:**
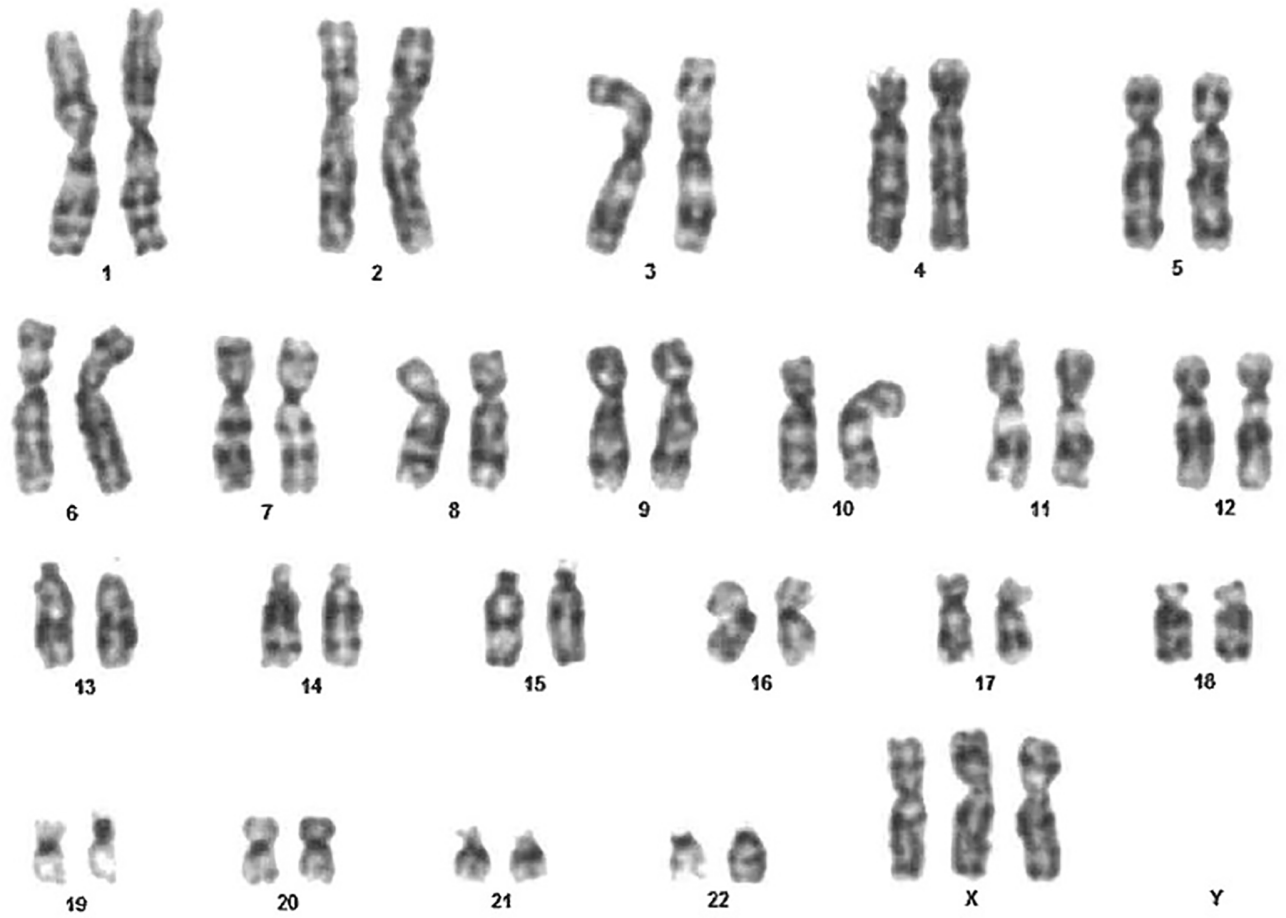
Postnatal karyotype of our patient.

A 6-year-old female presented to our hospital for recurrent upper respiratory tract infections and chronic diarrhea. The patient had a prenatal diagnosis of Trisomy X. A postnatal karyotype analysis of a peripheral blood sample of the girl revealed a karyotype of 47, XXX in all 100 metaphase cells analyzed, confirming the prenatal diagnosis of Trisomy X Syndrome (**Figure 1**).

The family history is notable for Type 2 Diabetes Mellitus and hypertension in both the father and paternal grandfather. The mother has hypertension and microcytic anemia, while the maternal grandfather has a Hashimoto thyroiditis.

Additionally, the patient’s 28-year-old sister has unspecified mandibular and ear malformations, and a maternal cousin is affected by cystic fibrosis. There is no family history of immunodeficiencies, allergies or early-age infections. Consanguinity and miscarriages are denied.

The pregnancy was uneventful aside from the karyotype finding of an additional X chromosome and was regularly monitored. The patient exhibited good cardiorespiratory adaptation at birth and had a normal neonatal period. She was breastfed and underwent regular weaning with a balanced diet. Growth and developmental milestones were appropriately achieved, and vaccinations are up to date.

Since the age of 6 months, the patient has had multiple episodes of bronchiolitis treated at home. From the first year of life, she experienced recurrent upper respiratory tract infections, characterized by rhinitis and productive cough, rarely associated with fever. She has been on antibiotic therapy every 20 days for about a year. She reported 2-3 episodes per year of bronchospasm treated with salbutamol nebulizer only. For approximately a year, the patient experienced recurrent episodes of watery diarrhea lasting 4-5 days every 20 days, accompanied by crampy abdominal pain. Between these episodes, she often experienced constipation and intestinal bloating. She also reported lower limb arthralgia during movement for about 15 days, which resolved spontaneously. Physical examination revealed a weight of 25 kg (86th percentile) and a height of 131.5 cm (99.6th percentile). These high percentiles are consistent with her Trisomy X diagnosis, and she is being followed by endocrinology for this genetic condition. Laboratory tests revealed a significant deficiency in IgA at 0,03 g/L (reference range: 0,34-3,48 g/L), which is a crucial finding given the patient’s recurrent infections. Other immunoglobulin levels were within normal ranges (**Table 1**).

**Table 1:** Blood test.

Test	Result	Reference Range
**RBC**	5.220.000	3.900.000-5.212.000/L
**Hb**	10.9	11.1 - 14.7 g/dL
**MCV**	67	76 - 90 fL
**IgA**	0.03	0,34 – 3,48 g/L
**IgG**	7,00	5,80 – 18,20 g/L
**IgM**	1,82	0,31 – 2,24 g/L
**IgE**	22	4-90 U/mL
**CD3**	1915	690 - 2540/mm³
**CD4**	1130	410 - 1590/mm³
**CD8**	675	190 - 1140/mm³
**CD19**	411	90 - 660/mm³
**NK**	85	90 - 590/mm³
**WBC**	7740	5000 - 13500/mm³
**N**	2310	1500 - 8000/mm³
**L**	4470	1500 - 5000/mm³
**M**	450	200 - 1300/mm³
**E**	340	0-1110/mm³
**B**	40	0-200/mm³
**PLT**	227.000	170.000 – 450,000/mm³
**FC during diarrhea**	347.52	<50 mg/kg
**FC symptom-free**	Negative	<50 mg/kg
**Anti-tTG IgA**	<2	<18 UI/ml
**Anti-tTG IgG**	3.1	<29 UI/ml
**Celiac genetic**	Absent	HLA-DQ2/HLA-DQ8 Present
**TSH**	1.33	0.45 - 5.2 µUI/mL
**FT3**	4.3	2.7 - 5.2 pg/mL
**FT4**	1.37	0.8 - 1.9 ng/dL
**Anti-TPO**	22	0 - 34 UI/mL
**Anti- Tg**	18.14	0 - 115 UI/mL
**Rubella IgG**	55.3	>10 U/mL
**Anti-HBs IgG**	<2.00	>10.00 UI/L
**Measles IgG**	>300	>16.5 mUI/mL
**Chickenpox IgG**	900	> 165 UI/mL
**Tetanus IgG**	2.23	>0.1 UI/mL
**Diphtheria IgG**	1.12	>0.1 UI/mL

RBC, Red Blood Cells; Hb, Hemoglobin; MCV, Mean Corpuscular Volume; Ig, Immunoglobulin; CD, Cluster of Differentiation; NK, Natural Killer cells; WBC, White Blood Cells; N, Neutrophils; L, Lymphocytes; M, Monocytes; E, Eosinophils; B, Basophils; PLT, Platelets; FC, Fecal Calprotectin; Anti-TTG, TSH, Thyroid-Stimulating Hormone; FT3, Free Triiodothyronine; FT4, Free Thyroxine; Anti-TPO, Anti-Thyroid Peroxidase Antibodies; HB, Hepatitis B.

The complete blood count was normal, showing microcytic anemia, which is most likely indicative of thalassemia minor. Lymphocyte subsets showed normal counts for CD3, CD4, CD8, CD19, and NK cells: liver and renal function tests were also within normal limits, suggesting no underlying hepatic or renal pathology. The patient’s total IgE and specific IgE levels for common food allergens were negative, and elevated calprotectin was noted during diarrheal episodes, though it returned to normal during symptom-free periods. Skin prick test was positive for Artemisa, but she did not suffer from allergic symptoms correlated with this allergen.

Screening for celiac disease was negative, including the absence of HLA-DQ2/DQ8 alleles, and thyroid function tests along with autoantibodies were negative. Notably, there was a lack of response to HBV vaccination, whereas antibodies for rubella, measles, chickenpox, diphtheria and tetanus were present (**Table 1**).

Imaging studies included an abdominal ultrasound, which was normal, and a pelvic ultrasound that revealed normal utero-ovarian structure with some microfollicula in the ovaries.

Bone age X-ray showed disharmonies among the various bone segments, particularly pseudo epiphyses at the bases of the second and fifth metacarpals, suggesting a minor growth delay, consistent with Trisomy X. The patient was advised to increase hydration and dietary fiber intake.

She was started on a treatment regimen for chronic constipation and instructed to follow up with gastroenterology and endocrinology. The patient has been followed up for 18 months, during which her immunological and clinical status has been regularly monitored. Immunological follow-up was planned to monitor her sDIgA and evaluate her response to vaccinations: a boost for HBV was indicated.

## Discussion

This case of a 6-year-old female with Trisomy X and SIgAD offers a unique perspective on the intricate relationship between the X chromosome and immune regulation.

The X chromosome plays a crucial role in immune regulation, as evidenced by various studies. In Trisomy X, the overexpression of certain genes that escape X-inactivation might contribute to immune anomalies. For instance, the *FOXP3* gene, located on the X chromosome, is essential for the function and maintenance of regulatory T cells, which are critical for immune tolerance. Dysregulation of such genes could lead to immune malfunctions, manifesting as immunodeficiencies like SIgAD (5, 9, 10).

The genetic cause of SIgAD is unknown, but evidence suggests a genetic predisposition based on familial clustering and association with known genetic loci. While the inheritance pattern is unclear, a family history of sIgAD or CVID increases the risk (11, 12).

Associations between sIgAD and MHC class I, II, and III haplotypes, particularly the extended MHC haplotype HLA A1, B8, DR3, and DQ2 (8.1 haplotype), have been noted (13). Other risk haplotypes include HLA-DR7, DQ2, DR1, and DQ5, while DR15 and DQ6 confer protection (14, 15).

Genetic variants in *TNFRSF13B* (TACI) were linked to sIgAD and CVID, but are now seen as disease-modifying rather than disease-causing (16–19). Non-HLA genes associated with sIgAD include IFIH1 and CLEC16A, both linked to autoimmune diseases like type 1 diabetes (T1D) and multiple sclerosis (20–23). A genome-wide study identified variants in *PVT1, ATG13-AMBRA1, AHI1, ICOS*, and *CLTA4*, indicating a complex genetic network influencing sIgAD (24, 25).

While many genes associated with sIgAD are located on various chromosomes, conditions like Triple X syndrome may indirectly influence immune function through epigenetic mechanisms or alterations in the regulation of immunity-related genes present on the X chromosome, such as *CD40LG* (Xq26.3), *CXCR3* (Xq13.1), *CXorf21* (Xp22.2), *IRAK1* (Xq28), *TLR7* (Xp22.2), *FOXP3* (Xp11.23), *GPR173* (Xq28), *MECP2* (Xq28), *PRPS2* (Xp22.31), *WAS* (Xp11.23), *BTK* (Xq21.33), *MAGT1* (Xq21.1), and *XIAP* (Xq25) (26). In the current scientific literature, there is no substantial evidence linking sIgAD to the X chromosome.

The article by Sills et al. (1978) describes a patient with Trisomy X, who presented with immunoglobulin deficiency and epilepsy, marking the unique documented case report linking Trisomy X to significant immune dysfunction (2). In this case, the patient exhibited hypogammaglobulinemia, characterized by significantly low levels of immunoglobulins, particularly IgA (0.07 g/L). The patient, despite being born following a normal pregnancy and making normal developmental progress, began to experience recurrent respiratory infections and seizures starting at around eight years old. Detailed immunological investigations revealed a severe deficiency in B lymphocytes, leading to the diagnosis of common variable immunodeficiency (CVID) (2).

Similar immune dysregulation is observed in other conditions involving X chromosome anomalies. Klinefelter Syndrome (47, XXY) and Turner Syndrome (45, X0) both demonstrate how variations in the number of X chromosomes can lead to immune system irregularities. Klinefelter Syndrome, characterized by an extra X chromosome, is associated with a higher prevalence of autoimmune diseases such as systemic lupus erythematosus (SLE) and other immune disorders, highlighting the impact of an additional X chromosome on immune function (27) (Silva et al., 2020). Turner Syndrome, with a single X chromosome, often presents with decreased levels of IgG and IgM, increased IgA, and reduced levels of circulating T- and B-lymphocytes, although findings are not always consistent (28).

The gene dosage effect is another potential mechanism. Research on the immunological aspects of these conditions reveals a broader pattern of immune dysregulation linked to X chromosome variations. For example, studies have shown that individuals with an extra X chromosome exhibit variations in serum levels of immunoglobulins, particularly IgM, suggesting a gene-dosage effect that influences immune function (2, 8). Additionally, the presence of autoimmune diseases and immunodeficiencies in these patients supports the hypothesis that an additional X chromosome disrupts the delicate balance of immune regulation (4, 29).

Epigenetic modifications also play a role in gene expression and immune function. The additional X chromosome in Trisomy X might lead to aberrant methylation patterns or histone modifications, affecting the expression of key immune regulatory genes. These epigenetic changes can alter the immune response, contributing to conditions like SIgAD. Research indicates that epigenetic regulation of the immune system is crucial, and disruptions can lead to various immunodeficiencies and autoimmune diseases (30).

Nuclear factor kappa-light-chain-enhancer of activated B cells (NF-κB) is a transcription factor that is essential in controlling the immune response, which includes the production of antibodies (31). Activating NF-κB, requires multiple essential steps and molecules, such as the NF-κB, essential modulator (*NEMO*) which encoded by *IKBKG* (Xq28) (32).

NEMO is essential for the activation of the IκB kinase (IKK) comple and is involved in B cell proliferation, differentiation, and survival (29). NF-κB, regulates the expression of various cytokines and signaling molecules essential for B cell function, such as BAFF (B-cell activating factor) and APRIL (a proliferation-inducing ligand). The survival and differentiation of plasma cells into antibody-secreting cells is enhanced by these cytokines’ activation of NF-κB, through their receptors on B cells (31).

Overexpression of X-linked genes, such as IKBKG (NEMO), could be caused by the presence of an extra X chromosome in individuals with triple X syndrome. An abnormal activation of the NF-κB, pathway may be caused by this overexpression, disrupting the delicate balance necessary for proper immune function. Such dysregulation could impair B cell activation and antibody production, possibly contributing to conditions like SIgAD.

The progression from SIgAD to CVID in patients with Trisomy X could indicate a complex interplay between genetic predisposition and immune system dysregulation. The overlap of these conditions underscores the necessity of monitoring and early detection to manage potential complications (4).

The patient’s poor response to certain vaccinations, such as HBV, and the frequent infections further indicate compromised humoral immunity. The lack of IgA, critical for mucosal immunity, makes her more susceptible to respiratory and gastrointestinal infections, exacerbated by her Trisomy X condition. Primary immunodeficiencies associated with chromosomal aberrations, including Trisomy X, are increasingly recognized. The European Society for Immunodeficiencies (ESID) has highlighted the importance of immunological investigations in patients with chromosomal anomalies and recurrent infections to identify potential immunodeficiencies that can be specifically treated (29).

Additionally, genetic studies have identified several X-linked loci associated with SLE, such as *TMEM187*, *IRAK1, MECP2, TLR7*, and *GPR173*, underscoring the significance of X-linked genetic variations in autoimmune disease susceptibility (27). The increased prevalence of trisomy X in SLE patients supports the hypothesis that an additional X chromosome contributes to the pathogenesis of autoimmune diseases (33).

Several immunodeficiencies associated with hypogammaglobulinemia result from mutations on the X chromosome, highlighting its crucial role in immune function. These conditions are predominantly found in males due to their single X chromosome, where a mutation can have a pronounced effect. However, females can be affected if they carry mutations on both X chromosomes or through skewed X-inactivation.

Wiskott-Aldrich Syndrome (WAS) is caused by mutations in the *WAS* gene (Xp11.23), which encodes the WASP protein. This syndrome presents with thrombocytopenia, eczema, recurrent infections, and often low levels of IgA​ and IgG (34)​. X-linked Agammaglobulinemia (XLA), due to mutations in the BTK gene, prevents the development of mature B cells. This leads to significantly reduced levels of immunoglobulins, including IgA, making patients prone to frequent bacterial infections​ (35). X-linked Severe Combined Immunodeficiency (X-SCID) involves mutations in the *IL2RG* gene, essential for T, B, and NK cell function. This condition leads to deficiencies in various immunoglobulins, including IgA​ (36). X-linked Hyper IgM Syndrome is caused by mutations in the *CD40LG* gene, disrupting normal immunoglobulin class switching. Patients have high levels of IgM and low levels of other immunoglobulins, such as IgA​ (37). X-linked Lymphoproliferative Syndrome (XLP) results from mutations in the *SH2D1A* gene, affecting the regulation of immune responses to viral infections and often leading to reduced levels of immunoglobulins (38). X-linked Immunodeficiency with Magnesium Defect, Epstein-Barr Virus Infection, and Neoplasia (XMEN) syndrome is due to mutations in the *MAGT1* gene, leading to defective magnesium transport, impaired T-cell function, and low IgA levels​ (39)​.

**Figure 2:**
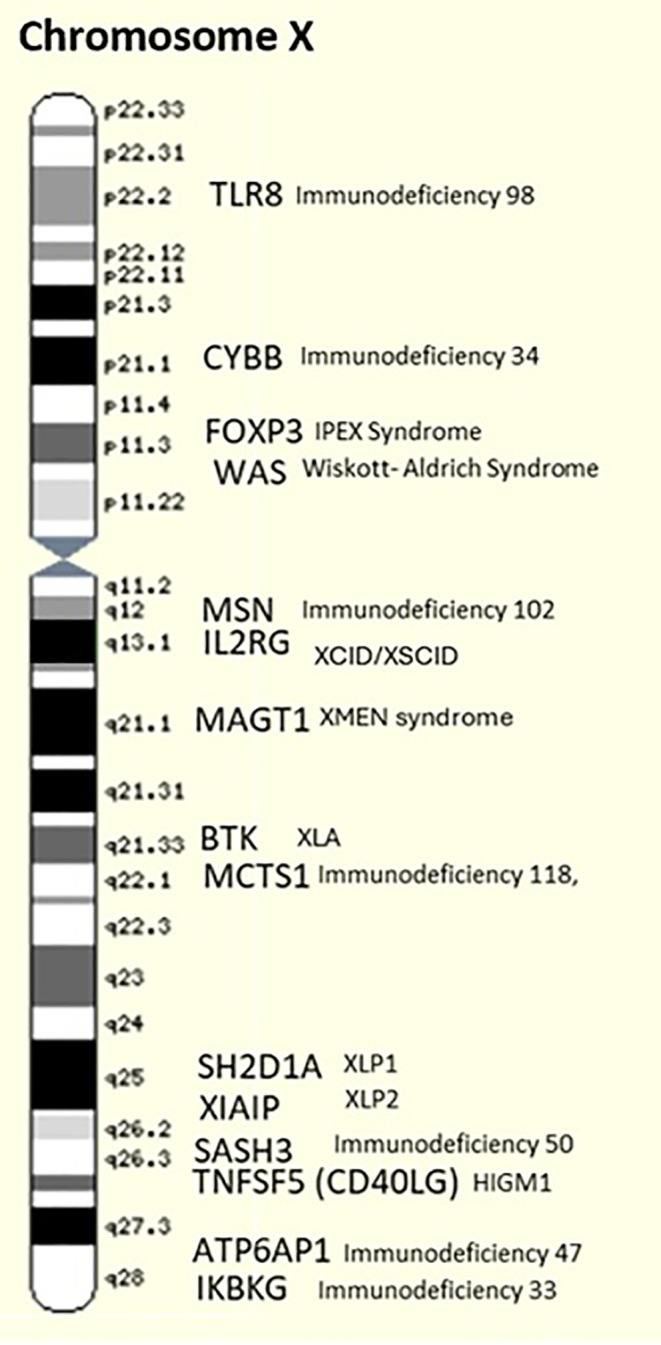
X chromosome gene map showing immunodeficiency-related genes and associated syndromes.

X-linked Inhibitor of Apoptosis (XIAP) Deficiency is caused by mutations in the *XIAP* gene, impairing apoptosis regulation, and leading to immune dysregulation and reduced IgA levels​ (40). Some other genes that have been identified to be related to X-linked immunodeficiencies are mentioned in **Table 2** and showed in **Figure 2**.

**Table 2:** X-linked immunodeficiencies.

X-linked immunodeficiencies	Gene/Locus	Gene/Locus MIM number
Wiskott-Aldrich Syndrome (WAS)	*WAS*	300392
X-linked Agammaglobulinemia (XLA)	*BTK*	300300
X-linked Severe Combined Immunodeficiency (X-SCID)	*IL2RG*	*308380*
Combined Immunodeficiency, X-Linked (XCID)	*IL2RG*	*308380*
Immunodeficiency With Hyper-IgM, Type 1 (HIGM1)	*TNFSF5* (CD40LG)	300386
Lymphoproliferative Syndrome, X-Linked, 1 (XLP1)	*SH2D1A*	300490
X-linked Immunodeficiency with Magnesium Defect, Epstein-Barr Virus Infection, and Neoplasia (XMEN) syndrome	*MAGT1*	300715
X-linked Inhibitor of Apoptosis (XIAP) Deficiency, Lymphoproliferative syndrome, X-linked, 2	*XIAP*	300079
Immunodeficiency 33, invasive pneumococcal disease, recurrent isolated, 2, formerly	*IKBKG*	300248
Immune dysregulation, Polyendocrinopathy, Enteropathy, X-linked (IPEX) Syndrome	FOXP3	300292
Immunodeficiency 34, mycobacteriosis, X-linked	*CYBB*	300481
Immunodeficiency 47, Congenital Disorder Of Glycosylation, Type IIs	*ATP6AP1*	300197
Immunodeficiency 50	*MSN*	309845
Immunodeficiency 98 with autoinflammation, X-linked	*TLR8*	300366
Immunodeficiency 102	*SASH3*	300441
Immunodeficiency 118, Mycobacteriosis	*MCTS1*	300587

These immunodeficiencies underscore the essential role of the X chromosome in maintaining a robust immune system. The significant number of genes on the X chromosome involved in immunoglobulin production necessitates consideration of potential immune dysfunctions in patients with X chromosome aneuploidies, such as Triple X syndrome. In these cases, the presence of an extra X chromosome can lead to altered gene dosage, which may affect immune regulation and function. Though typically females with Triple X syndrome do not exhibit severe immune deficiencies, the potential for subtle immunological abnormalities, including IgA deficiency, should not be overlooked due to the complex interactions of X-linked genes and their impact on immune responses​. In the end, X chromosome aneuploidies, like Trisomy X, may enhance the likelihood of germline and somatic mutations within lymphocytes, potentially exacerbating immune dysregulation due to both increased gene dosage and disrupted genomic stability (41).

## Conclusion

This case study underscores the complex interplay between chromosomal anomalies such as Trisomy X and selective immunodeficiencies like SIgAD. The findings suggest a possible link between X chromosome’ s alteration and immune dysregulation, possibly through mechanisms involving gene dosage effects or disruption of normal gene silencing. Importantly, the lack of typical genetic markers for SIgAD in this patient with Trisomy X highlights the potential for novel genetic pathways influencing IgA production, yet to be fully understood. The case emphasizes the necessity for vigilant genetic and immunological screening in patients diagnosed with chromosomal abnormalities, particularly those presenting with recurrent infections or other signs of immune dysfunction. Understanding the specific genetic contributions in these cases can lead to more precise, tailored therapies that address the underlying causes of the disease rather than just managing symptoms. Future research should focus on exploring the specific genes on the X chromosome that are involved in immune regulation and how their expression might be altered in Trisomy X. Such studies will not only deepen our understanding of the genetic basis of immunodeficiencies in chromosomal disorders but also potentially reveal new targets for therapeutic intervention, improving outcomes for these patients. Understanding the genetic basis of immune dysregulation in X chromosome aneuploidies will provide broader insights into the complex interplay between genetics and immunity. Additionally, serological testing for Pneumococcus and Haemophilus, as well as a post-booster evaluation for HBV response, has been requested; however, these results are currently pending due to the parents’ decision to delay testing, considering the patient’s stable clinical condition. While analyses such as regulatory T cell profiling, PD-1 expression as a marker of immunosenescence, and detailed evaluation of B and T cell subpopulations would provide valuable insights into the potential mechanisms of immune dysregulation, it is important to note that the patient’s SigAD limits the clinical indications for such advanced investigations. Future follow-up will incorporate the pending results and consider additional analyses, including markers of immunosenescence, if clinically indicated, to further delineate the patient’s immune profile.

## Data Availability

The raw data supporting the conclusions of this article will be made available by the authors, without undue reservation.
